# miR-23a binds to p53 and enhances its association with miR-128 promoter

**DOI:** 10.1038/srep16422

**Published:** 2015-11-10

**Authors:** Jincheng Li, Lynn Htet Htet Aung, Bo Long, Danian Qin, Shejuan An, Peifeng Li

**Affiliations:** 1 Department of Physiology, Shantou University School of Medicine, Shantou 515031, China; 2 Department of Microbiology and Immunology, College of Medicine, University of Illinois at Chicago, Chicago, IL 60612, USA; 3 Central Research Laboratory, Peking Union Medical College Hospital, Peking Union Medical College & Chinese Academy of Medical Sciences, Beijing, 100730, China

## Abstract

Apoptosis plays an important role in cardiac pathology, but the molecular mechanism by which apoptosis regulated remains largely elusive. Here, we report that miR-23a promotes the apoptotic effect of p53 in cardiomyocytes. Our results showed that miR-23a promotes apoptosis induced by oxidative stress. In exploring the molecular mechanism by which miR-23a promotes apoptosis, we found that it sensitized the effect of p53 on miR-128 regulation. It promoted the association of p53 to the promoter region of miR-128, and enhanced the transcriptional activation of p53 on miR-128 expression. miR-128 can downregulate prohibitin expression, and subsequently promote apoptosis. Our data provides novel evidence revealing that miR-23a can stimulate transcriptional activity of p53.

Apoptosis plays a critical role in both normal and disease conditions of cardiovascular system^1^,^2^,^3^. For example, focal apoptosis is the major contributor to the development of cardiac outflow tracts, cardiac valves, conducting system, and the developing coronary vasculature^1^,^2^,^3^. On the other hand, apoptosis is also a major pathology of cardiomyocyte loss during myocardial ischemia, infarction, reperfusion, and heart failure^4^,^5^,^6^,^7^,^8^. Therefore, the identification of the molecular pathways regulating apoptosis will bring new therapeutic insights to apoptotic-related cardiac diseases.

p53 is a well documented apoptotic signal in various cell types. It initiates apoptosis through intrinsic pathways by transcriptionally activating the pro-apoptotic protein expression^9^,^10^,^11^,^12^,^13^,^14^. Under oxidative stress, activation of p53 regulates transcription of several genes through miRNAs, which in turn leads to either upregulation or downregulation of pro and anti-apoptotic protein expression^9^,^15^. For example, under cell-stress, p53 induces the expression of miR-34 a/b/c and miR-145 to down-regulate the anti-apoptotic protein expression and thus promotes the cells to undergo apoptosis^9^,^16^,^17^. To date, growing lists of p53 downstream miRNA targets have been identified, however, the ability of miRNAs in regulating the effects of p53 remains unknown.

miRNAs are a class of small non-coding RNAs and have been evidenced to have a pivotal role in regulating gene expression in cardiomyocytes^18^,^19^,^20^. Several recent studies on miRNAs renovate our understanding on the regulation of apoptosis. Differential miRNAs play distinct roles in apoptosis by regulating either pro-apoptotic or anti-apoptotic pathway^9^,^15^,^16^. For example, miR-1 participates in the initiation of apoptosis^21^, whereas miR-21 is able to inhibit apoptosis^22^,^23^. They mediate post-transcriptional gene silencing, typically by binding to 3′UTR region of mRNAs^18^,^24^,^25^. However, it is unknown whether miRNA has a direct regulatory effect on transcriptional activity of transcriptional factors.

Prohibitin is a mitochondrial protein^26^, and has been reported to be related to apoptosis^27^,^28^,^29^. Enforced expression of prohibitin can inhibit serum withdraw- and staurosporin-induced apoptosis^28^. Prohibitin controls cell proliferation and apoptosis by regulating cristae morphogenesis in mitochondria^29^. Prohibitin is abundantly expressed in the heart^26^, but its function in cardiomyocytes remains to be elucidated.

Our study found that cardiomyocytes underwent apoptosis in response to hydrogen peroxide or doxorubicin treatment. Oxidative stress stimulation upregulated the miR-23a expression. This enhanced the association of p53 to the promoter region of miR-128 and transcriptionally activated the miR-128 expression. Strikingly, miR-128 suppressed the expression of prohibitin and thus promoting apoptosis. Our data provide novel evidence showing that miR-23a, p53, miR-128 and prohibitin form an axis in regulating apoptotic machinery.

## Results

### miR-23a participates in the regulation of apoptosis

**Figure 1:**
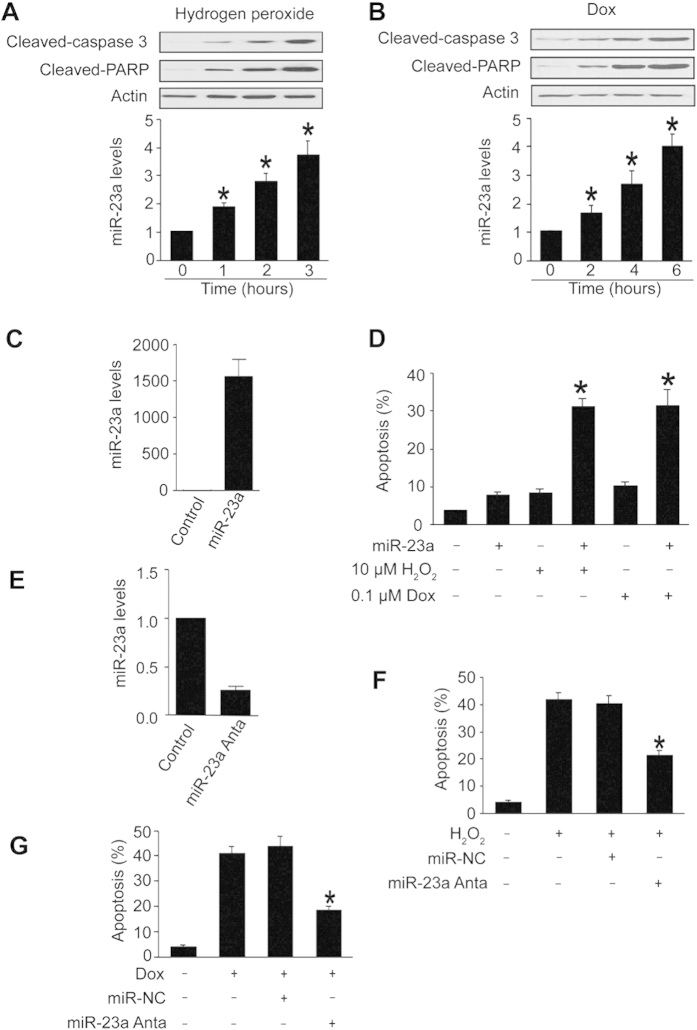
miR-23a promotes apoptosis. (**A**,**B**, upper panel) The levels of cleaved caspase-3 and cleaved PARP were elevated in response to hydrogen peroxide and doxorubicin treatment. Cardiomyocytes were treated with 100 μM hydrogen peroxide (H_2_O_2_, A) or 1 μM doxorubicin (Dox, B), at indicated time and cleaved caspase-3 and cleaved PARP were analysed by immunoblot. The total amount of β-actin served as internal control. (**A**,**B**, lower panel) miR-23a was elevated in response to apoptotic stimuli. Cardiomyocytes were treated with 100 μM hydrogen peroxide (**A**) or 1 μM doxorubicin (**B**), and harvested at the indicated time for the detection of miR-23a by qRT-PCR. *p < 0.05 vs control. (**C**) Cardiomyocytes were transfected with miR-23a mimics and the expression of miR-23a were detected by qRT-PCR. (**D**) miR-23a overexpression sensitized cells to undergoing apoptosis. Cardiomyocytes were transfected with miR-23a mimics, and then treated with hydrogen peroxide or doxorubicin. Apoptosis was analyzed by TUNEL assay. *p < 0.05 vs hydrogen peroxide alone or doxorubicin alone. (**E**) Cardiomyocytes were transfected with miR-23a antogomir and the expression of miR-23a were detected by qRT-PCR. (**F**,**G**) Knockdown of miR-23a reduced apoptosis. Cardiomyocytes were transfected with miR-23a antagomir (miR-23a anta) or the negative control (miR-NC), and then treated with 100 μM hydrogen peroxide (**F**) or 1 μM doxorubicin (**G**). Apoptosis was analyzed by TUNEL assay. *p < 0.05 vs hydrogen peroxide alone or doxorubicin alone.

We studied the role of miR-23a in apoptosis. Cardiomyocytes underwent apoptosis upon treatment with hydrogen peroxide (Fig. 1A) or doxorubicin (Fig. 1B). miR-23a was upregulated in response to hydrogen peroxide (Fig. 1A) or doxorubicin treatment (Fig. 1B). Overexpression of miR-23a (Fig. 1C) itself induced no apoptosis (Fig. 1D). Intriguingly, overexpression of miR-23a sensitized the cells to undergo apoptosis induced by hydrogen peroxide or doxorubicin treatment (Fig. 1D). Knockdown of miR-23a (Fig. 1E) attenuated apoptosis induced by hydrogen peroxide (Fig. 1F) or doxorubicin treatment (Fig. 1G). These findings suggest that miR-23a is able to promote apoptosis.

### miR-23a binds to p53

**Figure 2:**
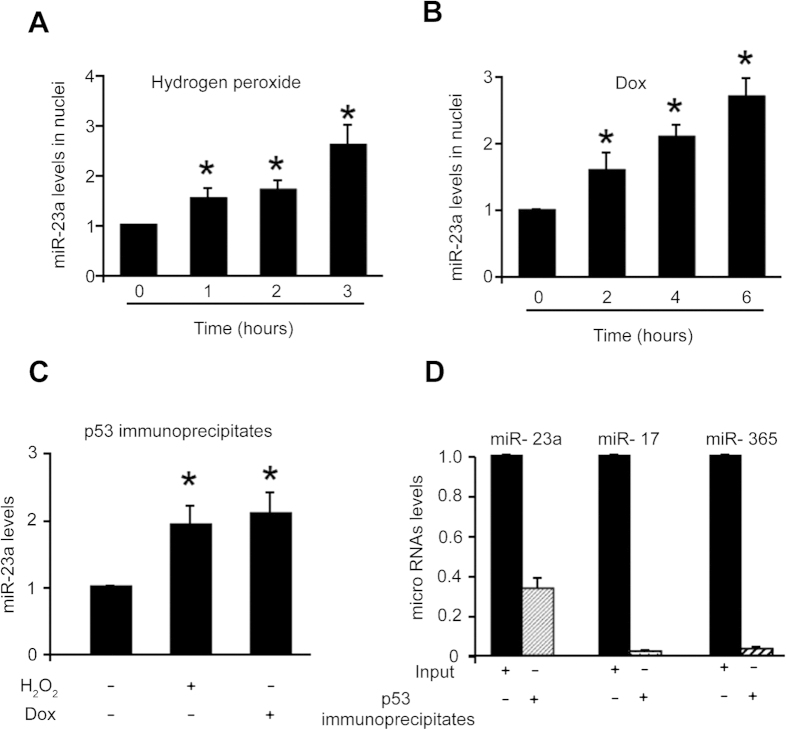
miR-23a binds to p53. (**A,B**) miR-23a is elevated in the cell nuclei. Cardiomyocytes were treated with 100 μM hydrogen peroxide (**A**) or 1 μM doxorubicin (**B**), and harvested at the indicated time for the isolation cell nuclei. miR-23a was analyzed by qRT-PCR. *p < 0.05 vs control. (**C**). miR-23a binds to p53. Cells were treated with 100 μM hydrogen peroxide or 1 μM doxorubicin. Cell nuclei were prepared, and immunoprecipitation was performed with the anti-p53 antibody. miR-23a was analyzed by qRT-PCR. *p < 0.05 vs control. (**D**) p53 and miR-23a association is specific. miR-23a, miR-17 or miR-365 were analyzed in the immunoprecipitates of p53 by qRT-PCR.

To understand the molecular mechanism by which miR-23a regulates apoptosis, we analyzed the levels of miR-23a in cell nuclei. Our results showed that the levels of miR-23a in the nuclei were elevated upon treatment with hydrogen peroxide (Fig. 2A) or doxorubicin (Fig. 2B). As p53 is well-known apoptosis inducer, we explored the relationship between p53 and miR-23a. We isolated cell nuclei, pulled down p53 by immunoprecipitation and found that miR-23a associate with p53 protein (Fig. 2C).

To know if the association of p53 with miR-23a is specific, we transfected the cells with the mimics of miR-23a, while miR-17 and miR-365 were randomly chosen as controls. We observed that a substantial amount of miR-23a but not miR-17 and miR-365 bound to p53, suggesting that their association is specific (Fig. 2D).

### miR-23a can promote the apoptotic effect of p53

**Figure 3:**
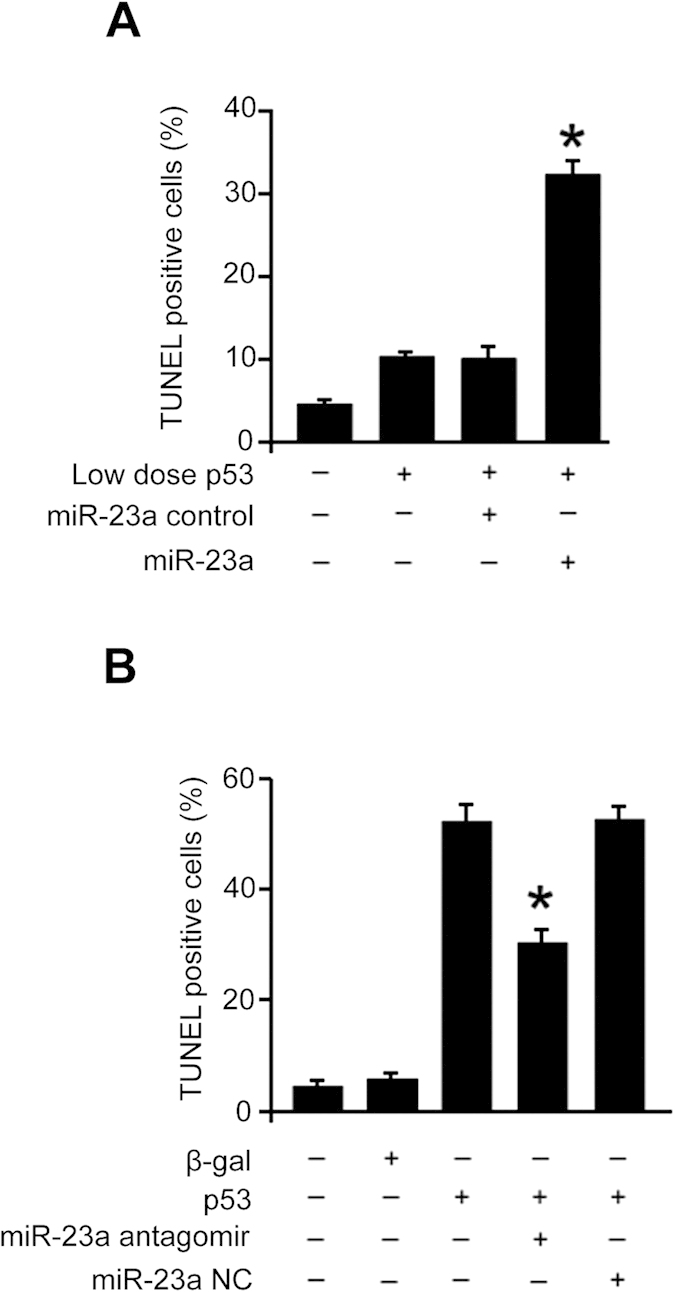
miR-23a can promote the apoptotic effect of p53. (**A**) miR-23a overexpression sensitizes p53 to induce apoptosis. The cells were infected with adenoviral p53 at a moi of 5, and then transfected with miR-23a mimics or its control (miR-23a control). Apoptosis was analyzed with the TUNEL assay. *p < 0.05 vs p53 alone. (**B**) Knockdown of miR-23a reduces apoptosis induced by p53. The cells were transfected with miR-23a antagomir or antagomir negative control (miR-23a NC), and then infected with adenoviral p53 at a moi of 60. Apoptosis was analyzed with the TUNEL assay 48 hours after infection. *p < 0.05 vs p53 alone.

In the functional analysis, we observed that a low dose of p53 induced less than 15% of cells to undergoing apoptosis. However, the same dose of p53 induced more than 30% of the cells to undergo apoptosis in the presence of miR-23a (Fig. 3A). To know if endogenous miR-23a plays a functional role, miR-23a was knocked down, and knockdown of miR-23a could reduce apoptosis induced by p53 (Fig. 3B). These data indicate that miR-23a can promote the apoptotic effect of p53.

### p53 associates with miR-128 promoter and transcriptionally stimulates its expression

**Figure 4:**
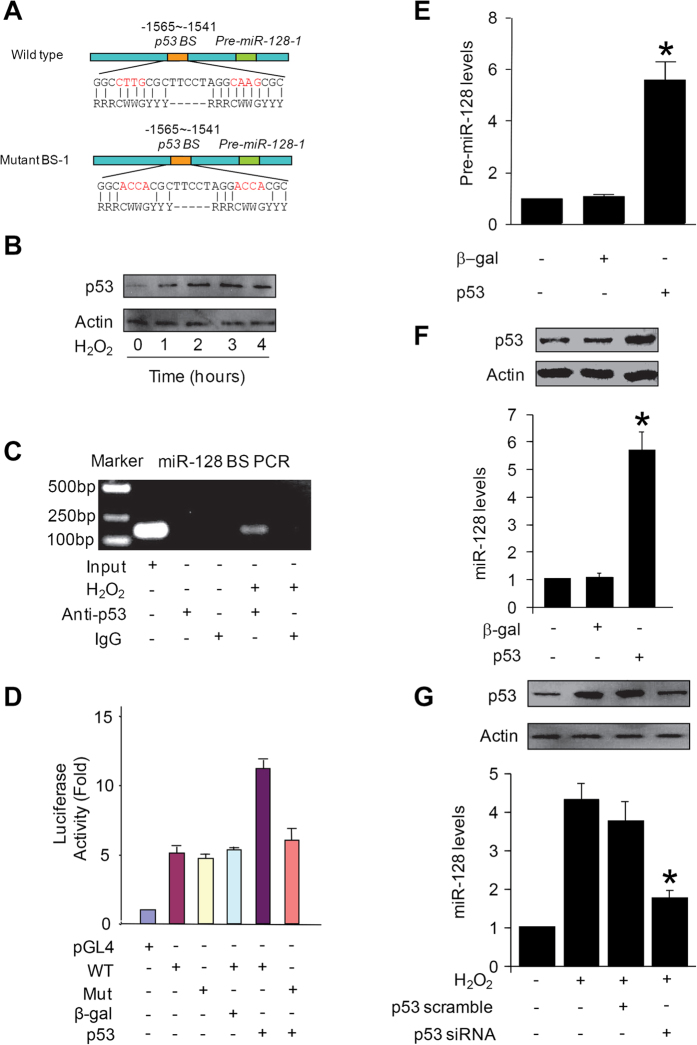
p53 associates with miR-128 promoter and transcriptionally stimulates its expression. (**A**) miR-128-1 promoter region contains a potential p53 binding site. The p53 binding site is composed of two 5′-RRRCWWGYYY-3′ sequences spaced by 0–21 base pairs. R is a purine, Y is a pyrimidine, W is either adenine or thymine, G is guanine and C is cytosine. The sequence was then mutated to disrupt p53 binding to the miR-128-1 promoter sequence. (**B**) Hydrogen peroxide induces an upregulation of p53. Cardiomyocytes were treated with 100 μM hydrogen peroxide, and harvested at the indicated time for the analysis of p53 by immunoblot. The total amount of β-actin served as internal control. (**C**) ChIP analysis of p53 binding to the promoter of miR-128-1 in cardiomyocyte treated with hydrogen peroxide. Cardiomyocyte were treated with hydrogen peroxide and were collected for ChIP assay. IgG was served as negative control. (**D**) Luciferase activity measured from cardiomyocytes infected with adenoviruses expressing p53 or β-galactosidase (β-gal) and transfected with empty vector (pGL4) or with constructs containing the wild-type (WT) miR-128-1 promoter or the miR-128-1 promoter mutated at putative p53 binding sites (Mut). (**E** and **F**) Enforced expression of p53 induces an elevation of pre-miR-128 (**E**) and miR-128 levels (**F**). Cardiomyocytes were infected with adenoviral p53 or β-gal. Pre-miR-128 (**E**) and miR-128 (**F**, lower panel) were detected by qRT-PCR. The results were normalized to that of U6. *p < 0.05, vs control. (**G**) Knockdown of p53 reduces miR-128 levels upon treatment with hydrogen peroxide. Cardiomyocytes were infected with adenoviral p53 siRNA (p53-siRNA) or its scramble form (p53-scramble). 24 hours after infection, cells were with 100 μM hydrogen peroxide. miR-128 was detected by qRT-PCR (**G**, lower panel). The results were normalized to that of U6. *p < 0.05, vs hydrogen peroxide alone. p53 were analyzed by immunoblot (**F** and **G**, upper panel). The total amount of β -actin served as internal control.

To search for the downstream targets of p53, we analyzed the promoter region of miR-128, and observed that miR-128 promoter regions contain the consensus p53 binding sites (Fig. 4A). Hydrogen peroxide induces an upregulation of p53 (Fig. 4B), and an association of p53 with miR-128 promoter (Fig. 4C). Luciferase assay demonstrated that p53 stimulates the promoter activity of the wild type miR-128 but not that of the mutated form (Fig. 4D).

We further tested whether p53 can regulate miR-128 expression levels. Enforced expression of p53 elevated Pre-miR-128 (Fig. 4E) and mature miR-128 levels (Fig. 4F). Knockdown of p53 reduced miR-128 levels upon hydrogen peroxide treatment (Fig. 4G). These results suggest that p53 can transcriptionally promote miR-128 expression.

### Prohibitin is a target of miR-128

**Figure 5:**
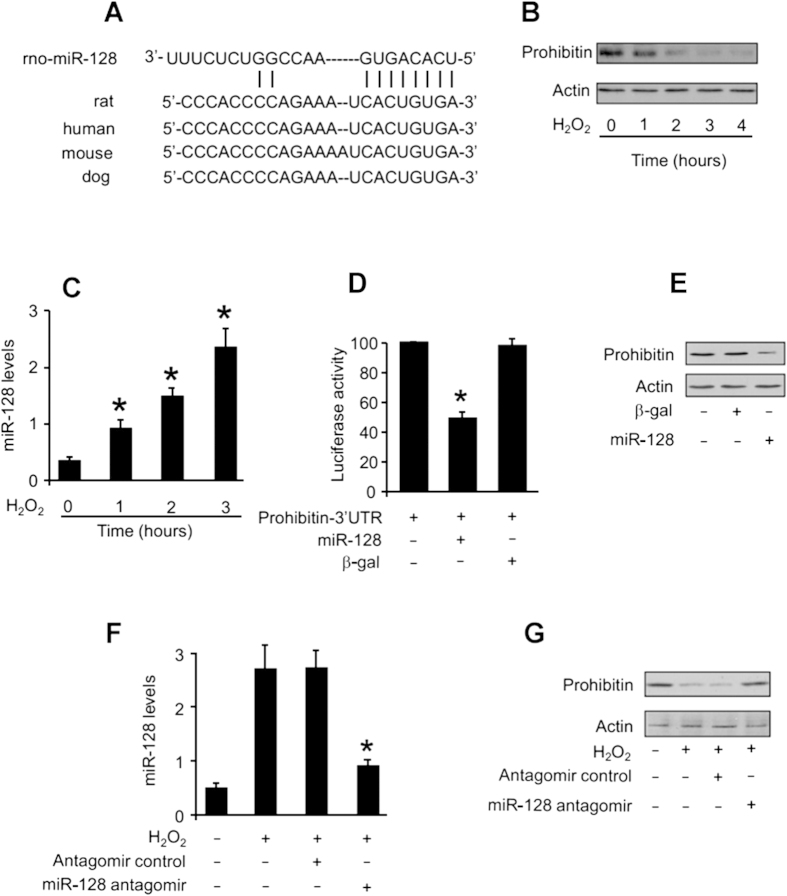
Prohibitin is a target of miR-128. (**A**) Prohibitin is a potential target of miR-128. miR-128 targeting site in the 3′UTR of prohibitin. (**B**) Prohibitin levels were reduced upon treatment with hydrogen peroxide. Cardiomyocytes were treated with 100 μM hydrogen peroxide, and harvested at the indicated time for the detection of prohibitin by immunoblotting. The total amount of β-actin served as internal control. (**C**) miR-128 levels were increased upon treatment with hydrogen peroxide. Cardiomyocytes were treated with 100 μM hydrogen peroxide, and harvested at the indicated time for the detection of miR-128 by qRT-PCR. The results were normalized to that of U6. *p < 0.05, vs control. (**D**) miR-128 inhibits the translational activity of prohibitin-3′UTR. HEK293 cells were infected with adenoviral constructs of miR-128 or β-galactosidase (β-gal). 24 h after infection, cells were transfected with the prohinitin-3′UTR luciferase construct. (**E**) Enforced expression of miR-128 reduces endogenous prohibitin levels. Cardiomyocytes were infected with adenoviral constructs of miR-128 or β-galactosidase (β-gal), and harvested 48 h after infection for the detection of prohibitin by immunoblotting. The total amount of β-actin served as internal control. (**F**) Knockdown of miR-128 by its antagomir. Cardiomyocytes were transfected with miR-128 antagomir or the antagomir control, and then treated with 100 μM hydrogen peroxide. 3 h after hydrogen peroxide treatment miR-128 was analyzed by qRT-PCR. *p < 0.05, vs control. (**G**) Knockdown of miR-128 attenuates the reduction of prohibitin levels. Cardiomyocytes were treated as described for (**F**). Prohibitin was analyzed by immunoblotting and the total amount of β-actin served as internal control.

We attempted to explore the molecular mechanism by which miR-128 exerts its effect. miRNAs have been shown to regulate protein expression levels. We analyzed the potential candidates of prohibitin using the program of target scan (http://www.targetscan.org), and miR-128 is a conserved miRNA of prohibitin (Fig. 5A). To understand whether prohibitin is involved in the apoptotic program of reactive oxygen species, we detected its levels in cardiomyocytes upon treatment with hydrogen peroxide. Prohibitin levels were reduced in response to the treatment with hydrogen peroxide (Fig. 5B). These results suggest that prohibitin can be a target of reactive oxygen species.

We tested whether miR-128 is responsible for the alterations of prohibitin under stress conditions. An elevation in miR-128 could be observed in cells treated with hydrogen peroxide (Fig. 5C). Thus, miR-128 is altered under oxidative stress condition. To understand whether prohibitin is a direct target of miR-128, we first tested whether miR-128 can influence prohibitin translational activity. Luciferase assay revealed that miR-128 could inhibit the translational activity of prohibitin (Fig. 5D). We next tested whether miR-128 is able to influence prohibitin expression. Enforced expression of miR-128 led to a reduction in endogenous prohibitin levels (Fig. 5E). We finally tested whether miR-128 participates in the regulation of prohibitin upon stimulation with oxidative stress. Administration of the miR-128 antagomir resulted in a reduction in miR-128 levels (Fig. 5F). Knockdown of miR-128 by its antagomir could attenuate the reduction in prohibitin levels in cells treated with hydrogen peroxide (Fig. 5G). These results suggest that miR-128 is able to target prohibitin.

### miR-128 regulates apoptosis through prohibitin

**Figure 6:**
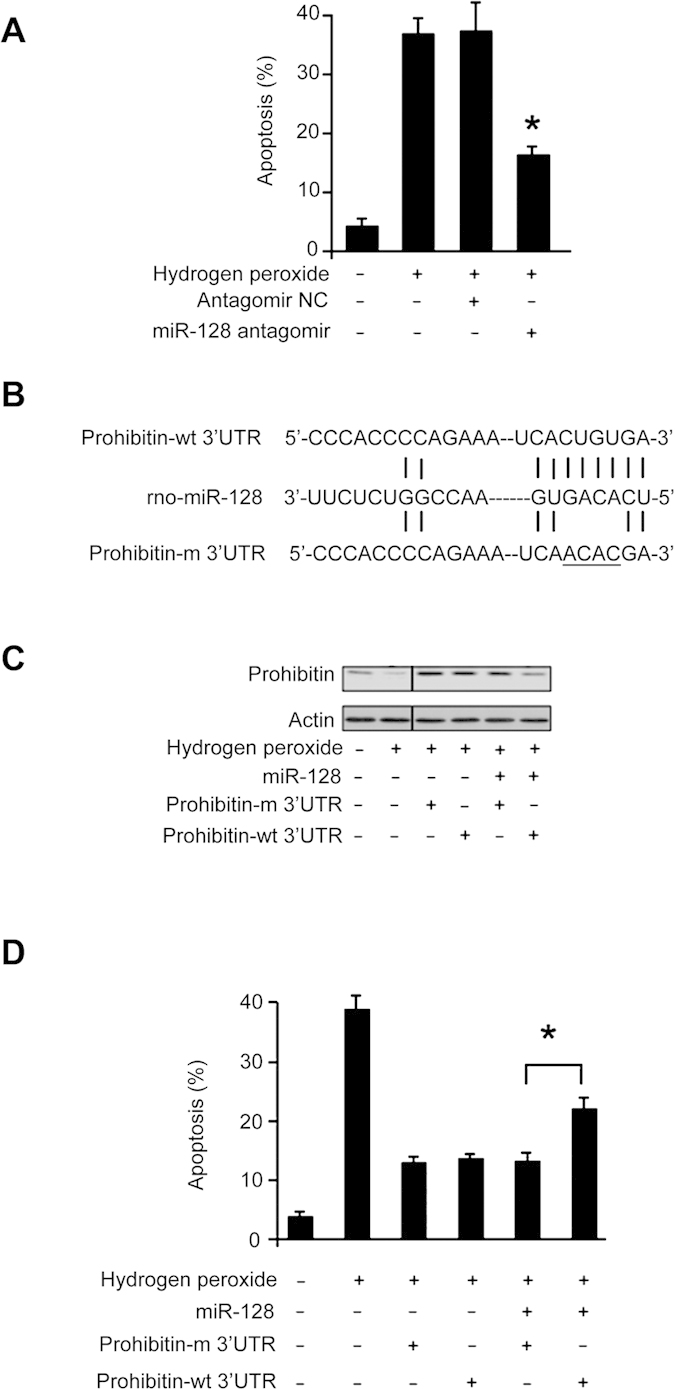
miR-128 regulates apoptosis through prohibitin. (**A**) Knockdown of miR-128 could attenuate apoptosis. Cardiomyocytes were transfected with miR-128 antagomir or the antagomir negative control (Antagomir-NC). 24 hours after transfection, cells were treated with 100 μM hydrogen peroxide. Apoptosis was analyzed by the TUNEL assay. *p < 0.05 vs hydrogen peroxide alone. (**B**) Constructs of prohibitin with wild type 3′UTR (prohibitin-wt 3′UTR) or mutated 3′UTR (prohibitin-m 3′UTR, the mutations are underlined). (**C**) miR-128 significantly reduces the expression levels of prohibitin with wild type 3′UTR but not with mutated 3′UTR. Cardiomyocytes were infected with the adenoviral construct of miR-128, along with the construct of prohibitin-wt 3′UTR or prohibitin-m 3′UTR. 24 h after infection, cells were treated with 100 μM hydrogen peroxide. Prohibitin levels were analyzed by immunoblotting and the total amount of β-actin served as internal control. (**D**) Prohibitin with mutated 3′UTR can more efficiently inhibit apoptosis than that with wild type 3′UTR in the presence of miR-128. Cardiomyocytes were treated as described for (**C**). Apoptosis was analyzed by the TUNEL assay. *p < 0.05.

The involvement of miR-128 in regulating prohibitin led us to consider whether it participates in the regulation of apoptosis. Knockdown of miR-128 could attenuate apoptosis induced by hydrogen peroxide treatment (Fig. 6A). We tested whether prohibitin can influence the ability of miR-128 to regulate apoptosis. We produced the wild type (wt) 3′UTR of prohibitin, and its mutated form in which mutations were introduced into the binding sites of miR-128 (Fig. 6B). Enforced expression of miR-128 could attenuate the expression of prohibitin with wt-3′UTR. However, a significant level of prohibitin still retained in cells expressing prohibitin with the mutated 3′UTR (Fig. 6C). Concomitantly, prohibitin with the mutated 3′UTR could be more effective than that with wt-3′UTR in antagonizing apoptosis (Fig. 6D) in the presence of miR-128. Taken together, it appears that miR-128 controls cell death through prohibitin.

### miR-23a requires p53 and miR-128 to regulate apoptosis

**Figure 7:**
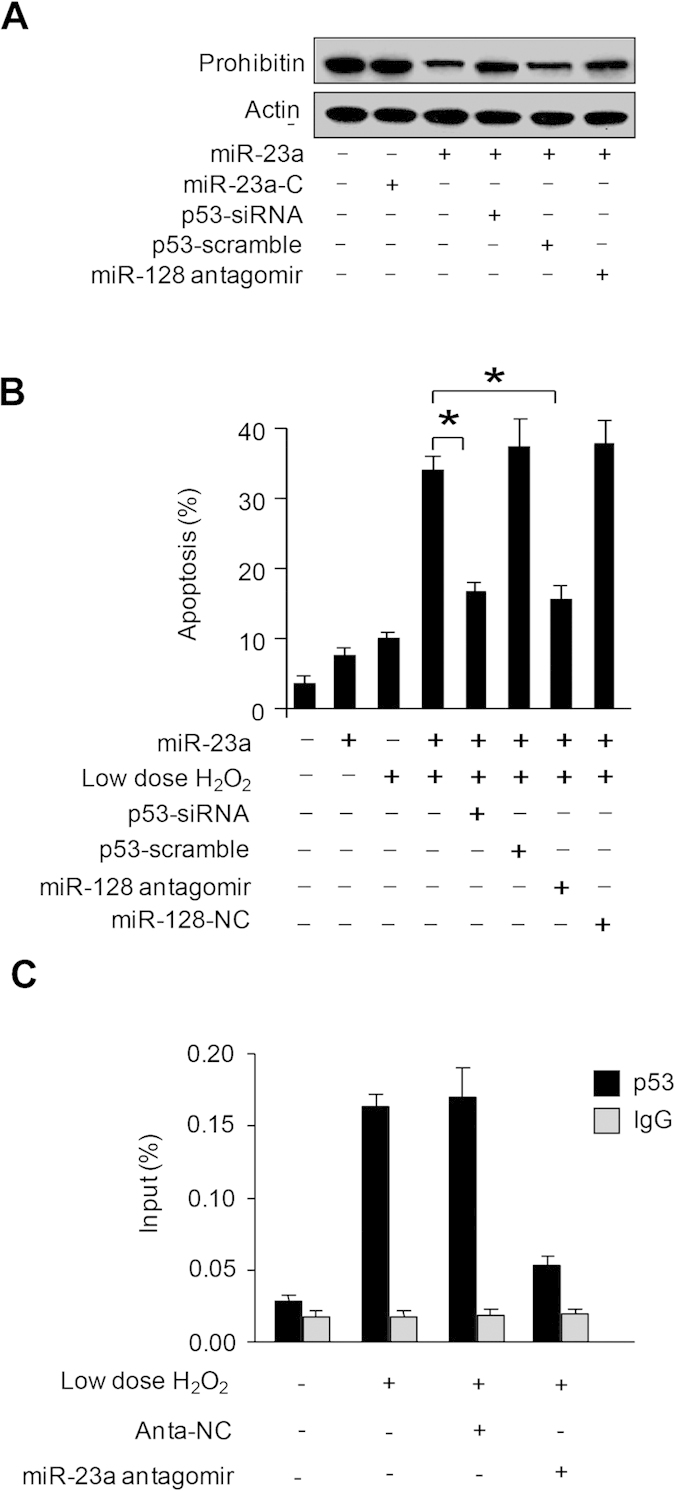
miR-23a requires p53 and miR-128 to regulate apoptosis. (**A**) p53 and miR-128 affect miR-23a in controlling prohibitin expression. Cardiomyocytes were infected with adenoviral p53 siRNA (p53-siRNA) or its scramble form (p53-scramble). 24 hours after infection, cells were transfected with miR-23a mimic (miR-23a), or miR-23a mimic control (miR-23a-C), or miR-128 antagomirs. Prohibitin levels were analyzed by immunoblotting and the total amount of β-actin served as internal control. (**B**) p53 and miR-128 affect miR-23a function in controlling apoptosis. Cells were treated as describe in (**A**), and treated with 10 μM hydrogen peroxide. Apoptosis was analyzed by the TUNEL assay 12 hours after treatment. *p < 0.05. (**C**) Inhibition of miR-23a significantly diminished the binding of p53 to miR-128 promoter. Cardiomyocytes were treated with miR-23a antogomir and the cells were collected for ChIP-qPCR assays.

In the following experiments, we explored the downstream mediators of miR-23a in regulating apoptosis. The expression of prohibitin was downregulated by miR-23a, however, knockdown of either p53 or miR-128 attenuated the effect of miR-23a (Fig. 7A), suggesting that p53 and miR-128 convey the signal from miR-23a in controlling prohibitin expression. As shown in Fig. 7B, miR-23a overexpression or a low dose of hydrogen peroxide alone led to a less amount of cells to undergo apoptosis. But, in the presence of hydrogen peroxide, miR-23a overexpression was able to induce a substantial amount of apoptosis, and this effect was attenuated by knockdown of either p53 or miR-128. Further, the binding between p53 and miR-128 promoters is reduced when miR-23a is depleted by its antagomir (Fig. 7C and Supplementary Fig. S1). It demonstrate that miR-23a is essential for p53 transcriptional activity on miR-128 promoter. These results suggest that p53 and miR-128 are downstream mediators of miR-23a in conveying apoptotic signal.

## Discussion

The heart has a limited capacity for regeneration and repair; the loss of cardiomyocytes in cardiac diseases such as myocardial infarct and heart failure cannot be compensated by efficient cell proliferation. Thus, understanding the signalling pathways responsible for cardiac apoptosis is crucial to effectively prevent the excessive cell loss in those conditions^30^. Our present work reveals that miR-23a is able to promote apoptosis in cardiomyocytes induced by hydrogen peroxide and doxorubicin and it can be a functional regulator of p53 dependent apoptotic pathway. Furthermore, we found that p53 directly binds to miR-128 promoter and enhances miR-128 expression. miR-128 suppresses prohibitin expression and thus promotes the cells to undergo apoptosis. Our data for the first time demonstrates that miR-23a, p53, miR-128 and prohibitin could form an axis in controlling cardiomyocyte apoptosis.

p53 plays an essential role in regulating apoptosis. Reactive oxygen species and anoxia lead to the up-regulation of p53 expression^8^,^14^,^31^. It has been reported that p53 may use transcription-dependent pathways to initiate apoptosis^12^,^32^. The functional signal transduction circuit of p53 consists of the upstream mediators, the core regulation components and the downstream effectors^33^. It directly regulates multiple downstream mediators such as Bax, Bad, Apaf-1 and caspases to convey its death signal. Although extensive reports have been made on upstream and downstream mediators of p53, only a few studies have reported on how the transcriptional activity of p53 is regulated. Here we found that miR-23a can promote the transcriptional activity of p53 by enhancing p53 binding to the miR-128 promoter in cardiomyocyte upon oxidative stress stimulation.

Recently, miRNAs were discovered as regulators of gene expression and found to be related to multiple cardiac pathologies^18^,^19^,^24^,^25^. Various types of stress signals to the heart can activate signal-transduction pathways that lead to either the upregulation or down-regulation of specific miRNAs^19^,^34^,^35^. Therefore, identifying those miRNAs, which are involved in cardiomyocyte apoptosis cascades and characterizing their signalling pathway could promote new insight to the treatment of apoptosis related cardiac diseases. A growing number of functionally important miRNAs in apoptotic pathway has been discovered^18^,^36^,^37^,^38^. Recently, it has shown that miR-23a play an important role in cancer cell apoptosis by promoting tumor cell proliferation^39^,^40^ and invasion^41^. However, its role in oxidative stress-induced cardiomyocyte apoptosis is not yet known. Our study found that miR23a and miR-128 were up regulated upon oxidative stress induced by hydrogen peroxide and doxorubicin. Strikingly, miR-23a promoted the transcriptional activation of p53 on miR-128 expression. The results demonstrated that the association of p53 and miR-128 promoter occurred in the presence of miR-23a. miR-23a may associate with the promoter region of miR-128 and thus recruiting p53. However, this needs to be further investigated. The binding of p53 to miR-128 promotes the negative regulatory effect of miR128 on prohibitin expression, and thus led higher percentage of the cells to undergo apoptosis. Our finding suggests that p53 could be the down-stream mediator of miR-23a. However, previous study in human hepatic cancer cell found that p53 could function as an upstream regulator of miR-23a expression (rather than down-stream mediator) upon etoposide treatment. DNA damage in HCC upregulated p53 induced miR-23 expression, which in turn sensitized the cells to anti-tumor effect of etoposide, thus promoted the cancer cells to undergo etoposide-induced apoptosis^39^.

Prohibitin is abundantly expressed in the heart under physiologic conditions^26^, but its function in the heart remains largely unknown. Our present work revealed that prohibitin was able to inhibit apoptosis. Furthermore, we found that prohibitin expression levels were reduced in response to oxidative injury in the cellular model. This finding was in consistence with the previous study, which demonstrated that prohibitin was downregulated in the heart in response to ischemic injury^42^. However, there was also a report showing that prohibitin was upregulated in the heart under chronic restraint stress^43^. One explanation for such discrepancy could be the variation in employment of the different experimental models^42^,^43^. The upregulation of prohibitin can be a cellular protective response against chronic restraint stress damage, while the downregulation of prohibitin may be a result of irreversible injury.

In summary, we found that miR-23a, participates in the initiation of apoptosis through targeting p53 in cardiomyocytes experiencing oxidative stress stimulation. During oxidative stress, miR-23a induces a substantial numbers of cells to undergo apoptosis. This effect was attenuated by knockdown of either p53 or miR-128. These results suggest that p53 and miR-128 could be downstream mediators of miR-23a in conveying apoptotic signal (the schematic diagram showing overall signaling pathway of this study was described in supplementary Fig. S2). This provides important information in developing novel therapeutic approaches for apoptosis-related cardiac diseases such as myocardial infarction and heart failure.

## Methods

The study design was approved by the Institutional Board of Shantou University, School of Medicine. All experiments were preformed according to the protocol approved by the Animal Care Committee, Shantou University School of Medicine.

### Materials

Adenovirus β-galactosidase was as we described^44^. Adenoviral p53, adenoviral p53 siRNA and adenoviral p53 scramble were as we described^15^. Hydrogen peroxide and doxorubicin were obtained from Sigma-Aldrich, St. Louis, MO.

### Cardiomyocytes culture, treatment and apoptosis analysis

Neonatal rat cardiac cells were isolated from 2-day-old Sprague-Dawley rats and prepared as we described^15^. In brief, after dissection hearts were washed, minced in HEPES-buffered saline solution contained (in mM): 130 NaCl, 3 KCl, 1 NaH_2_PO_4_, 4 glucose and 20 HEPES (pH adjusted to 7.35 with NaOH). Tissues were then dispersed in a series of incubations at 37 °C in HEPES-buffered saline solution containing 1.2 mg/ml pancreatin and 0.14 mg/ml collagenase (Worthington). After centrifugation cells were re-suspended in Dulbecco’s modified Eagle medium/F-12 (GIBCO) containing 5% heat-inactivated horse serum, 0.1 mM ascorbate, insulin-transferring-sodium selenite media supplement, 100 U/ml penicillin, 100 μg/ml streptomycin, and 0.1 mM bromodeoxyuridine. The dissociated cells were pre-plated at 37 °C for 1 hour. The cells were then diluted to 1 × 10^6 ^cells/ml and plated in 10 μg/ml laminin-coated different culture dishes according to the specific experimental requirements. After 24 hours medium was changed by serum free medium. Cells were treated with hydrogen peroxide as we described^15^, or doxorubicin. For apoptosis analysis, the terminal deoxynucleotidyl transferase-mediated dUTP nick-end-labeling (TUNEL) kit (Clontech) was used according to the kit’s instructions. 200–300 cells were counted in 30–40 random fields in each group.

### Immunoblotting analysis

Immunoblotting was carried out as we previously described^44^. Cells were lysed for 1 h at 4 °C in a lysis buffer (20 mmol/L Tris pH 7.5, 2 mmol/L EDTA, 3 mmol/L EGTA, 2 mmol/L dithiothreitol (DTT), 250 mmol/L sucrose, 0.1 mmol/L phenylmethylsulfonyl fluoride, 1% Triton X-100) containing a protease inhibitor cocktail. Samples were subjected to 12% SDS-PAGE and transferred to nitrocellulose membranes. Equal protein loading was controlled by Ponceau Red staining of membranes. Blots were probed using the primary antibodies. The anti-cleaved PARP and the anti-cleaved caspase-3 were from Abcam. The anti-prohibitin antibody and the anti-α-actin antibody were from Santa Cruz Biotechnology. The anti-p53 antibody was from Calbiochem. After four times washing with PBS Tween-20, the horseradish peroxidase-conjugated secondary antibodies were added. Antigen-antibody complexes were visualized by enhanced chemiluminescence. The total amount of β-actin served as internal control.

### Preparation of nuclei

Nuclei of cardiomyocytes were prepared as described^45^. In brief, cells were washed twice with ice-cold hypotonic buffer (20 mM potassium-HEPES [pH 7.8], 5 mM potassium acetate, 0.5 mM MgCl_2_, and 0.5 mM DTT). Cells were allowed to swell for 10 min in 10 ml hypotonic buffer per plate. Cells were scraped off the plates and disrupted with 25 strokes in a dounce homogenizer using a loose-fitting pestle. Nuclei were pelleted at 4000 rpm for 5 min. Pelleted nuclei were resuspended and washed three times in phosphate-buffered saline and finally pelleted at 5000 rpm for 5 min.

### Immunoprecipitation

Immunoprecipitation was prepared as described^46^. The samples were precleared with 10% (vol/vol) Protein-A (Roche) for 1 h on a rocking platform. The anti-p53 antibody was added and rocked for 1 h. Immunoprecipitates were captured with 10% (vol/vol) Protein-A agarose for another hour. The agarose beads were spun down and washed three times with NET/NP40 buffer (150 mM NaCl, 2 mM EDTA, 50 mM Tris-HCl pH 7.5, 0.1% NP-40).

### Preparations of the luciferase construct of rat prohibitin 3′UTR

Prohibitin 3′UTR was amplified by PCR using the upstream primer 5′-GGCCAGCCAGGCCAGGGCCTC-3′, and the downstream primer 5′- CAGAATGGAGGCTCAAGGTC-3′. It was subcloned into the pGL3 vector (Promega) immediately downstream of the stop codon of the luciferase gene.

### Construction of mutated 3′UTR of prohibitin by site-directed mutagenesis

The introduction of mutations into the miR-128 binding site in the 3′UTR of prohibitin was performed using QuikChange II XL Site-Directed Mutagenesis Kit (Stratagene) according to the manufacturer's instructions. The construct was sequenced to check that only the desired mutations had been introduced.

Preparation of adenoviral miR-128 was synthesized by polymerase chain reaction using rat cardiomyocyte DNA as the template. The upstream primer was 5′-TTTCATTCTTGGGCTCTTTG-3′. The downstream primer was 5′-GAAGAGAAAGCAATAGCTAC-3′. It was cloned into the Adeno-X^TM^ Expression System (Clontech) according to the manufacturer's instructions.

### Adenovirus infection

Viruses were amplified in 293 cells and purified on a CsCl gradient. Cells were infected with the virus at the indicated multiplicity of infection (moi). After washing with PBS, culture medium was added and cells were cultured until the indicated time.

### Analysis of miR-23a, miR-17, miR-128, and miR-365 by quantitative real-time PCR (qRT-PCR)

These microRNAs' levels were measured by qRT-PCR using their TaqMan® MicroRNA Assays kits according to the manufacturer's instructions (Life Technologies). Total RNA was prepared by TRIzol reagent (Invitrogen). qRT-PCR was carried out in duplicate in a 7500 Fast Real-Time PCR System (Applied Biosystems) as we described^15^. The levels of these microRNAs analyzed by qRT-PCR were normalized to that of U6.

### Analysis of pre-miR-128 expression

For quantitative detection of pre-miR-128, qRT-PCR was designed according to the previously described method^47^,^48^. Briefly, total RNA was prepared by TRIzol reagent (Invitrogen). RNA was processed to reverse transcriptase reactions using reverse transcriptase kit (ReverTra Ace, Toyobo). Real time PCR using SYBR Green Realtime PCR Master Mix (ReverTra Ace, Toyobo) was carried out in triplicate in an in a 7500 Fast Real-Time PCR System (Applied Biosystems) according to the manufacturer’s instructions. The sequences of rat pre-miR-128 primers were Forward-1: 5′-CTTTCATTCTTGGGCTCTTTG-3′; Forward-2: 5′-TGAGCTGTTGGATTCGGGGCC-3′; Reverse: 5′-GAAGCAGCTGAAAAAGAGACC-3′.

### Chromatin immunoprecipitation analysis (ChIP) and ChIP-qPCR

ChIP assay was performed as described with modifications^49^. In brief, cells (0.5×10^7^) were washed with PBS and incubated for 10 min with 1% formaldehyde at room temperature. The cross-linking was quenched with 0.1 M glycine for 5 min. Cells were washed twice with PBS and lysed for 1 h at 4 °C in a lysis buffer. The cell lysates were sonicated into chromatin fragments with an average length of 500–800 bp as assessed by agarose gel electrophoresis. The samples were precleared with Protein-A agarose (Roche) for 1 h at 4 °C on a rocking platform, and 5 μg anti-p53 antibodies were added and rocked for overnight at 4 °C. Immunoprecipitates were captured with 10% (vol/vol) Protein-A agarose for 4 h. Before use, Protein-A agarose was blocked twice at 4 °C with salmon sperm DNA (1 μg ml^−1^), which had been sheared to a 500-bp length, and bovine serum albumin (1 μg ml^−1^) overnight. The final ChIP DNAs were then used as templates in PCR reactions, using primers that encompass p53-binding site of the rat miR-128 promoter. The oligonucleotides were as follows: forward: 5′-GCAGTGATGTGTAGGAATTGT-3′; reverse: 5′-TGAATTCCCACCTGTTCTATA-3′. The primers for detecting miR-128 promoter region without p53 binding domain were as follows: forward: 5'-GTGCTTTCTTGGGCCTGAAA-3'; reverse: 5'-AGAACCATAGATGGAAAAAC-3'.

### miR-128 promoter

The miR-128-1 promoter region was amplified from rat genomic DNA to generate wild type promoter. The forward primer was 5′-ATGGTCTGACTCCTAGGTGTCAG-3′. The reverse primer was 5′-GGAACAAGGCCCATTATTGTA-3′. The PCR product was cloned into the vector pGL4.17 (Promega). The introduction of mutations in the putative p53-binding site in the wild-type fragment was generated using QuikChange II XL Site-Directed Mutagenesis Kit (Stratagene). The construct was sequenced to check that only the desired mutations had been introduced.

### Transfection of microRNAs's mimics or antagomirs

miR-23a mimic, mimic negative control, miR-23a antagomir, miR-128 antagomir, and antagomir negative control were obtained from Life Technologies. Cells were transfected with mimics or antagomirs using Lipofectamine 2000 (Invitrogen) according to the manufacturer's instruction. miR-23a antagomir or the antagomir negative control were at a concentration of 70 nM. miR-23a mimic or the mimic negative control were at a concentration of 50 nM. miR-128 antagomir or the antagomir negative control were at a concentration of 100 nM.

### Luciferase assay for prohibitin 3′UTR

HEK293 cells were seeded in 12-well plates (5 × 10^4^ cells/well), and infected with adenoviral constructs of miR-128 or β-galactosidase at a moi of 10. 24 h after infection cells were transfected with the plasmid construct of prohibitin 3′UTR using the Effectene Transfection Kit (Qiagene). Each well contains 0.2 μg luciferase reporter plasmids, 5 ng SV-Renilla luciferase plasmids as the internal control. Cells were harvested 24 h after transfection for the detection of luciferase activity using the Dual Luciferase Reporter Assay kit (Promega) according to the manufacturer’s instructions. 20 μl of protein extracts were analyzed in a luminometer. Firefly luciferase activities were normalized to Renilla luciferase activity.

### Luciferase assay for miR-128-1 promoter

The miR-128-1 promoter luciferase assay was performed as described^49^. Briefly, cells were seeded in 24-well plates. They were transfected with the luciferase constructs using the Lipofectamine 2000 (Invitrogen). Each well contains 0.2 μg luciferase reporter plasmids, 2.5 ng Renilla luciferase plasmids as the internal control. After transfection for 24 h, cells were harvested for the detection of luciferase activity, using the Dual Luciferase Reporter Assay kit (Promega) according to the manufacturer’s instructions. A volume of 20 μl of protein extracts were analysed in a luminometer. Firefly luciferase activities were normalized to Renilla luciferase activity.

### Statistical analysis

Data are expressed as the mean ± s.e.m. of at least three independent experiments. We evaluated the data with Student’s t-test. We used a one-way analysis of variance for multiple comparisons. A value of p < 0.05 was considered significant.

## Additional Information

**How to cite this article**: Li, J. *et al.* miR-23a binds to p53 and enhances its association with miR-128 promoter. *Sci. Rep.* **5**, 16422; doi: 10.1038/srep16422 (2015).

## Supplementary Material

Supplementary Data
