# Supplementary material for: miR-23a binds to p53 and enhances its association with miR-128 promoter
Source: Sci Rep. 2015 Nov 10;5:16422. doi: 10.1038/srep16422 (PMC4639766; doi:10.1038/srep16422)

Supplementary material

miR-23a Binds to p53 and Enhances its Association with miR-128 Promoter

Jincheng Li[1*], Lynn Htet Htet Aung[2*], Bo Long [3*], Danian Qin[1], Shejuan An[2] and Peifeng Li[2]

[1] Department of Physiology, Shantou University School of Medicine, Shantou 515031, China

[2] Department of Microbiology and Immunology, College of Medicine, University of Illinois at Chicago, Chicago, IL 60612, USA

[3] Central Research Laboratory, Peking Union Medical College Hospital, Peking Union Medical College & Chinese Academy of Medical Sciences, Beijing, 100730, China

*These authors contributed equally to this work.

Supplementary figure legend

Supplementary figure S1. A control ChIP-qPCR assay was performed using primer encompassing miR-128 promoter region without p53 binding domain. Cardiomyocytes were treated with miR-23a antogomir and the cells were collected for ChIP-qPCR assays. miR-128 promoter without p53 binding site did not associate with p53 regardless of presence or absence of miR-23a antagomir.

Supplementary figure S2. The schematic diagram showing miR-23a promotes p53 transactivation on miR-128, which induces apoptosis by inhibiting prohibitin expression in oxidative stress-induced cardiomyocyte.

Supplementary figure S1

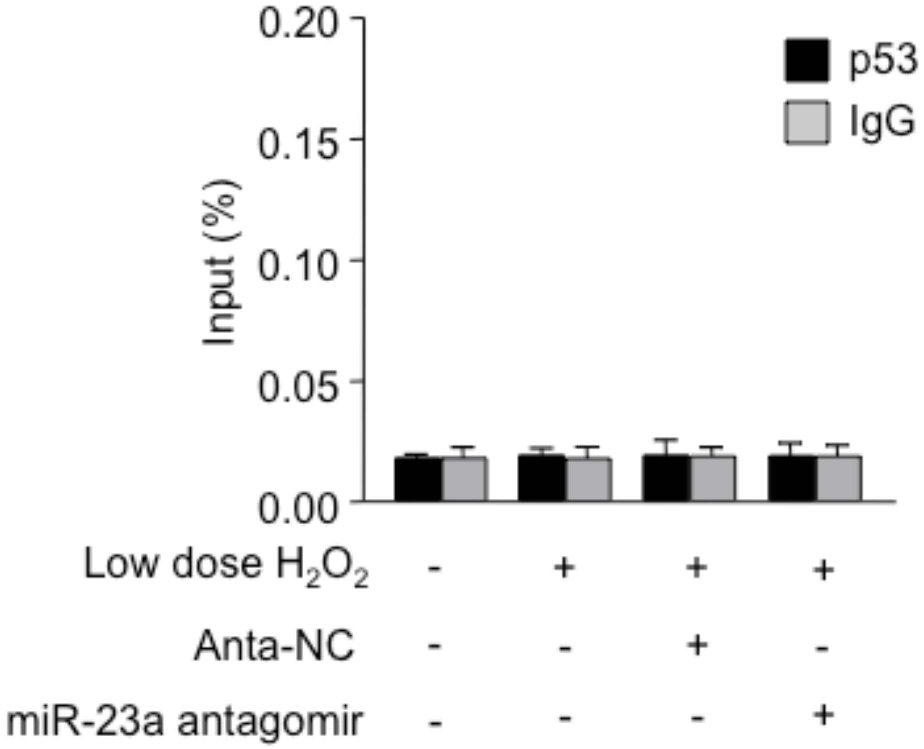

Supplementary figure S2

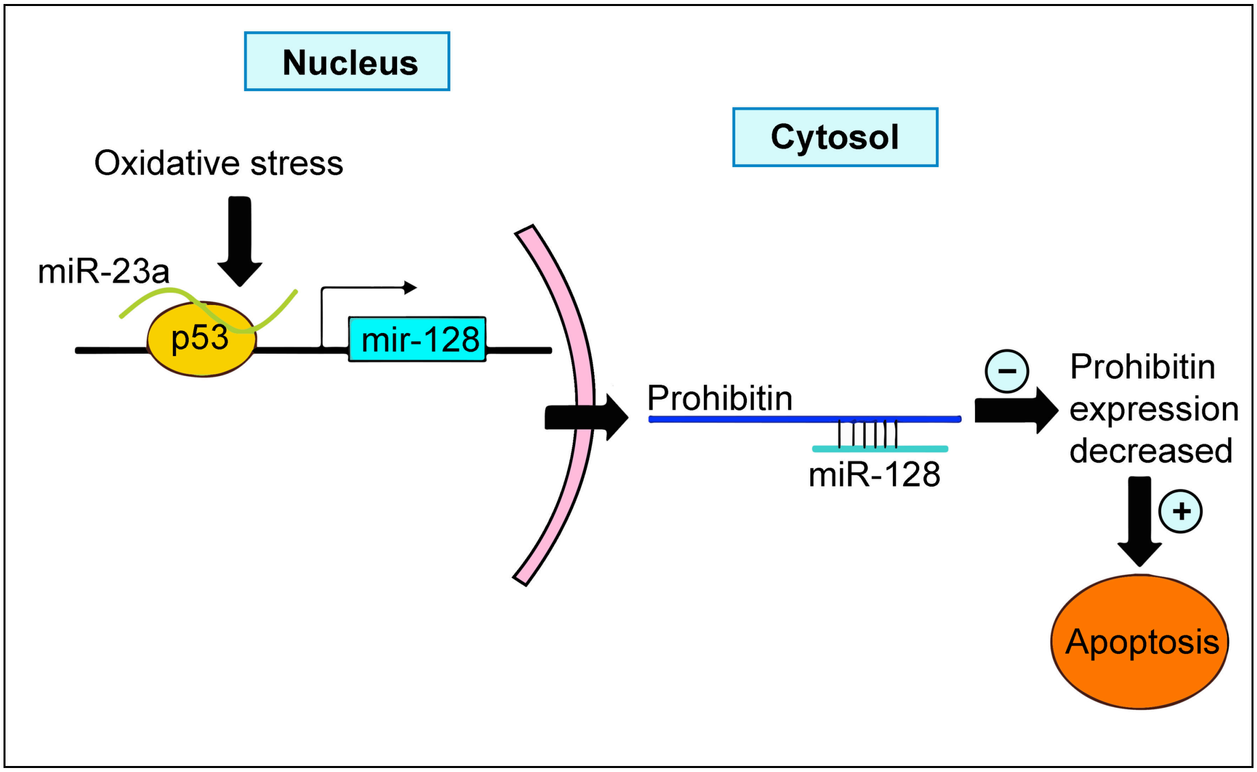

Supplement: Supplementary Data [file srep16422-s1.pdf]
